# Gene Expression Profiling of IL-17A-Treated Synovial Fibroblasts from the Human Temporomandibular Joint

**DOI:** 10.1155/2015/436067

**Published:** 2015-12-29

**Authors:** Toshio Hattori, Naomi Ogura, Miwa Akutsu, Mutsumi Kawashima, Suguru Watanabe, Ko Ito, Toshirou Kondoh

**Affiliations:** ^1^Department of Maxillofacial Surgery, Nihon University School of Dentistry at Matsudo, Matsudo, Chiba 271-8587, Japan; ^2^Research Institute of Oral Science, Nihon University School of Dentistry at Matsudo, Matsudo, Chiba 271-8587, Japan

## Abstract

Synovial fibroblasts contribute to the inflammatory temporomandibular joint under pathogenic stimuli. Synovial fibroblasts and T cells participate in the perpetuation of joint inflammation in a mutual activation feedback, via secretion of cytokines and chemokines that stimulate each other. IL-17 is an inflammatory cytokine produced primarily by Th17 cells which plays critical role in the pathogenesis of numerous autoimmune and inflammatory diseases. Here, we investigated the roles of IL-17A in temporomandibular joint disorders (TMD) using genome-wide analysis of synovial fibroblasts isolated from patients with TMD. IL-17 receptors were expressed in synovial fibroblasts as assessed using real-time PCR. Microarray analysis indicated that IL-17A treatment of synovial fibroblasts upregulated the expression of IL-6 and chemokines. Real-time PCR analysis showed that the gene expression of IL-6, CXCL1, IL-8, and CCL20 was significantly higher in IL-17A-treated synovial fibroblasts compared to nontreated controls. IL-6 protein production was increased by IL-17A in a time- and a dose-dependent manner. Additionally, IL-17A simulated IL-6 protein production in synovial fibroblasts samples isolated from three patients. Furthermore, signal inhibitor experiments indicated that IL-17-mediated induction of IL-6 was transduced via activation of NF*κ*B and phosphatidylinositol 3-kinase/Akt. These results suggest that IL-17A is associated with the inflammatory progression of TMD.

## 1. Introduction

The temporomandibular joint (TMJ) is one of the most complex and active joints in the human body, playing an important role in functions such as jaw motion speaking, chewing, and swallowing. Patients with temporomandibular disorders (TMD) most frequently present with pain, limited mandibular motion, and TMJ sounds. Inflammatory factors contribute to both inflammatory and degradative pathways associated with the progression of the pathological condition in the joints [1–3]. These inflammatory factors have been detected in the synovial fluids and/or tissues from patients with intracapsular pathological conditions of TMJ such as disc displacement (DD), internal derangement (ID), and/or osteoarthritis (OA) [1, 2].

Synovitis, an inflammatory disorder of the synovial membrane, frequently accompanies ID and/or OA in TMJ [4, 5] and has been suggested to be a key feature of intracapsular pathological conditions of TMJ [6]. The synovial membrane lines all of the intra-articular structures, except for the articular cartilage of the eminence, fossa and mandibular condyle, and the articular disc [7]. The lining layer of synovial tissue is composed of fibroblast-like cells and macrophage-like cells and overlies loose connective tissue of the synovial sublining that contains blood vessel sublining fibroblasts and leukocytes. In orthopedics, synovial fibroblasts that are producing a number of putative mediators of inflammation and tissue degradation [8–10] and other immune cells communicate with one another in a unique inflammatory microenvironment [9]. An understanding of the molecular mechanisms that underlie the activities of these factors may contribute to an understanding of the pathogenesis of TMD; however, little is known about the molecular mechanisms that underlie the development of the pathological condition in TMJ.

Interleukin- (IL-) 17 is secreted primarily by active Th17 cells, and IL-17s and IL-17 receptors play an important role in numerous autoimmune and inflammatory diseases [11, 12]. The IL-17 family consists of six family members of varying homology and function: IL-17A (commonly called IL-17), IL-17B, IL-17C, IL-17D, IL-17E (also called IL-25), and IL-17F [13, 14]. The IL-17 receptor (IL-17R) family includes five members (IL-17RA to IL-17RE) [13, 14]; both IL-17A and IL-17F bind to the same IL-17 receptor complex consisting of the receptor subunits IL-17RA and IL-17RC [13, 14]. IL-17 has been implicated in progression of arthritis in rheumatoid arthritis (RA) and OA. IL-17 was detectable in serum and knee synovial fluid samples from patients with OA and RA, and a positive association was found between the IL-17 concentration and the disease severity and/or activity [15, 16]. In vitro experiments with human cells identified IL-17A as a contributor to the promotion of synovial hyperplasia, synoviocyte invasion, cartilage degradation, and angiogenesis [17–20]. The pathogenic potential of IL-17A in inflammatory arthritis has also been reported in studies involving neutralization of IL-17A and in IL-17A-deficient mice [21].

Recently, IL-17 was also detected in synovial fluid from the TMJ with ID and OA [22]; however its role has not been studied in TMD. We isolated human synovial fibroblasts from the synovial tissue of patients with intracapsular pathological conditions of TMJ and then examined the gene expression profile of these cells when treated with IL-17A. The aim of this study was to investigate the roles of IL-17A in the pathogenesis of TMD.

## 2. Materials and Methods

### 2.1. Isolation and Culture of Synovial Fibroblasts

Human synovial tissue was obtained from three patients (TMJ1-3) who underwent arthroscopy for ID or open TMJ surgery for OA (TMJ1, female, age: 23 years, used for the oligonucleotide microarray analysis, real-time PCR, and ELISA. TMJ2, female, age: 26 years, used for ELISA. TMJ3, male, age 59 years, used for ELISA). All patients provided informed consent for the surgery and for the use of their tissue specimens for research purposes. The isolation of, primary culture of, and experimentation with synoviocytes were performed according to the guidelines established by the Institutional Review Board of Nihon University School of Dentistry at Matsudo (Ethics Committee Registration Number: EC10-037).

Human synovial fibroblasts isolated from the synovial tissues of patients with intracapsular pathological conditions of TMJ (synovial fibroblasts) were prepared using the outgrowth method previously reported by Ogura et al. [23]. In brief, synovial tissue samples were washed with phosphate-buffered saline (PBS), minced, placed in a 35 mm tissue culture dish, and covered with a sterilized glass coverslip. The culture medium used was Ham's F12 (Wako, Osaka, Japan) supplemented with 10% fetal bovine serum (FBS) (Cell Culture Technologies, Gravesano, Switzerland), 100 *μ*g/mL penicillin G (Meiji, Tokyo, Japan), 100 *μ*g/mL kanamycin sulfate (Meiji), and 250 ng/mL Fungizone (Gibco, Grand Island, NY, USA). The medium was changed twice per week. Confluent SFCs were detached with 0.025% trypsin (Gibco) and 0.02% EDTA in PBS and were then subcultured in Ham's F12 supplemented with 10% FBS and antibiotics. For the experiments, FLSs obtained from passages 6 to 8 were used.

### 2.2. Total RNA Extraction

Confluent-stage synovial fibroblasts were cultured in medium containing 2% FBS for 24 h and were then stimulated with or without 10 ng/mL IL-17A (PeproTech Inc., Rocky Hill, NJ, USA) for various lengths of time. Total cellular RNA from synovial fibroblasts was extracted using the RNeasy Mini Kit (Qiagen, Valencia, CA, USA) and was then stored at −80°C until use.

### 2.3. DNA Microarray Analysis

Total RNA samples from synovial fibroblasts treated with IL-17A (10 ng/mL) for 4 h and from untreated control samples were profiled on a SurePrint G3 Human Gene Expression 8x60K v2 Microarray (Agilent Technologies Inc., Santa Clara, CA, USA), according to Agilent protocols. The array was scanned using an Agilent DNA Microarray Scanner. Gene expression analysis of the DNA microarray was performed using GeneSpring GX software (Agilent). Data were normalized using raw data from each array as a reference. Changes in gene expression were determined by comparing the normalized intensities for untreated cells with those of IL-17A-treated cells. The microarray data have been deposited in the National Center for Biotechnology Information Gene Expression Omnibus (GEO Series GSE74668; http://www.ncbi.nlm.nih.gov/geo/).

### 2.4. Signaling Pathway Analysis

Biologically relevant pathways of IL-17A-responsive genes were constructed using Ingenuity Pathway Analysis (IPA) (Ingenuity Systems, http://www.ingenuity.com/, Redwood City, CA, USA). A dataset of the gene accession numbers and gene expression ratios (IL-17A-treated/control) of greater than 2-fold intensity as determined by the GeneSpring GX software program were uploaded into the IPA. Each gene identifier was mapped to its corresponding gene object in the Ingenuity Pathway Knowledge Base. The uploaded genes, called focus genes, were overlaid onto a global molecular network developed from information contained in the Ingenuity Pathway Knowledge Base. The analysis in IPA identifies relationships, mechanisms, functions, and pathways relevant to a dataset.

### 2.5. Real-Time Polymerase Chain Reaction (Real-Time PCR)

Complementary DNA was synthesized from total RNA using a GeneAmp RNA PCR Kit (Thermo Fisher Scientific Inc., Waltham, MA, USA). Real-time PCR was performed using a DyNAmo SYBR Green qPCR Kit (Thermo Fisher Scientific Inc.). The PCR mixture (20 *μ*L) contained 20 pmol forward and reverse primers and 2 *μ*L cDNA. Amplification was performed using a DNA Engine Opticon 1 (Bio-Rad, Hercules, CA, USA), with preheating at 95°C for 10 min, followed by 40 cycles of 94°C for 15 s, 55°C for 30 s, and 72°C for 30 s. The genes analyzed in this study were examined for their relative expression to their respective control by using the ΔΔC_T_ method [24]. All analyses were performed in five replicates, and the results were confirmed by five independent experiments.

PCR fragments were electrophoresed on 1.5% agarose gels, followed by staining with Midori Green Direct (NIPPON Genetics, Tokyo, Japan) and examination of fragment sizes. The primer sequences used for the real-time PCR analysis are shown in Table 1.

### 2.6. Enzyme Linked Immunosorbent Assay (ELISA)

Synovial fibroblasts were plated at a density of 5 × 10^4^ cells per well in 24-well plates with Ham's F12 medium containing 10% FBS. Confluent cells were cultured for 24 h in the same medium containing 2% FBS. After incubation with IL-17A for the appropriate length of time, culture supernatants were collected and stored at −80°C until use. The kinetics of IL-6 protein production was examined in control samples and in synovial fibroblasts incubated with IL-17A (10 ng/mL) for 4, 8, 12, and 24 h. To examine the dose dependency of IL-6 protein expression, the cells were treated with IL-17A at concentrations of 1, 10, and 50 ng/mL for 24 h. The IL-6 levels in the conditioned medium were measured using ELISA kit (R&D Systems, McKinley, MN, USA), according to the manufacturer's protocol. The ELISA experiments were independently performed six times.

### 2.7. Inhibition of IRAK 1/4, PI3K, TAK1, and IKK*β*

Synovial fibroblasts were plated at a density of 5 × 10^4^ cells per well in 24-well plates with Ham's F12 medium containing 10% FBS. Confluent cells were cultured for 24 h in medium containing 2% FBS. The inhibition experiments were performed using the following inhibitors: Interleukin-1 Receptor-Associated-Kinase-1/4 (IRAK-1/4) inhibitor (20 *μ*M) (Merck KGaA, Darmstadt, Germany), the phosphoinositide 3-kinase (PI3K) inhibitor LY294002 (20 *μ*M) (Merck KGaA), the transforming growth factor-*β*-activated kinase 1 (TAK1) inhibitor (5z)-7-Oxozeaenol (1 *μ*M) (Merck KGaA), and the inhibitor of the NF*κ*B kinase *β* subunit (IKK*β*) inhibitor PS-1145 (10 *μ*L) (Cayman Chemical, Ann Arbor, MI, USA). The cells were pretreated with the inhibitor reagents for 30 min, followed by incubation with IL-17 (10 ng/mL). After 8 h, the culture supernatants were collected and stored at −80°C until use. The inhibitor effect was calculated as 100 − [(IL-6 production with IL-17 in the presence of the inhibitor)/(IL-6 production with IL-17) × 100]. The IL-6 levels in the conditioned medium were measured using ELISA kit (R&D systems).

### 2.8. Statistical Analysis

The data are expressed as means ± standard deviations (SD) and were analyzed using one-way analysis of variance (ANOVA). Post hoc analyses were carried out using the Student-Newman-Keuls (SNK) Multiple Comparison Test. *P* < 0.05 and *P* < 0.01 were considered to indicate significant differences.

## 3. Results

### 3.1. Expression of IL-17 Receptor Family Members in Synovial Fibroblasts

**Figure 1:**
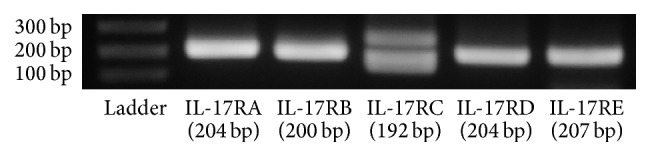
The expression of IL-17 receptors in synovial fibroblasts. IL-17RA, IL-17RB, IL-17RC, IL-17RD, and IL-17RE mRNA levels in synovial fibroblasts were analyzed using real-time PCR. The PCR products were electrophoresed through an agarose gel.

Before examination of the functional effects of IL-17A (which was used as a typical IL-17) in synovial fibroblasts, we first analyzed the expression of IL-17R family members A-E in synovial fibroblasts using real-time PCR. All IL-17R family members were expressed in synovial fibroblasts (Figure 1). IL-17A signals through a heterodimeric receptor complex composed of IL-17RA and IL-17RC [14]. These data therefore suggested that IL-17A signaling is transduced in synovial fibroblasts.

### 3.2. Microarray Analysis of Synovial Fibroblasts

**Figure 2:**
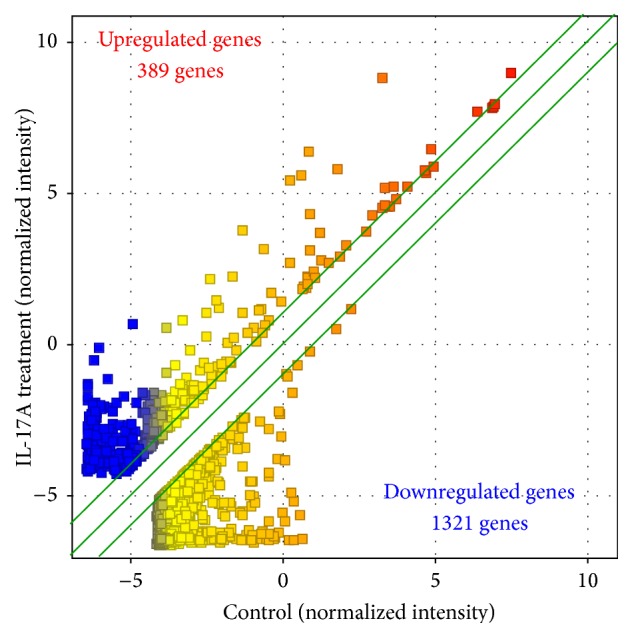
Scatter plots of microarray analysis. Of the 50,739 genes on the DNA microarray, the 27,583 genes that were expressed in synovial fibroblasts were compared between synovial fibroblasts treated with IL-17A and nontreated control. Of these 27,583 genes, 1,710 genes (389 upregulated genes and 1321 downregulated genes) showed a greater than twofold difference between IL-17A-treated and control cells.

We next analyzed the gene expression profiles of synovial fibroblasts that were treated with or without IL-17A to determine the mechanisms underlying its effects in pathological conditions of TMJ. Of the 50,739 genes on the DNA microarray, 27,583 genes were expressed in synovial fibroblasts, and the expression of these genes was compared between nontreated control cells and IL-17A-treated cells. Genes that showed a greater than twofold difference in expression between IL-17A-treated and control cells were further analyzed. A total of 1,710 genes showed greater than 2-fold changes in expression with IL-17 treatment; the expressions of 389 of these genes were upregulated, and the expressions of 1,321 of these genes were downregulated (Figure 2). The 1,710 IL-17-responsive genes were categorized based on the gene ontology of molecular function using GeneSpring software. Many upregulated genes were categorized functionally in the ligands of receptors such as chemokines, growth factors, and cytokines that are regulators associated with inflammation and immunity. In contrast, several downregulated genes were categorized as receptors for ligands (Table 2).

### 3.3. IL-17A Signaling Pathway Analysis

To investigate the existence of biologically relevant pathways for IL-17A-responsive genes in synovial fibroblasts, we uploaded a dataset of IL-17A-responsive genes containing gene identifiers and corresponding fold change values obtained by the DNA microarray analysis into the IPA system as focus genes. The 1,710 IL-17A-responsive genes were categorized based on gene ontology (data not shown). The most highly related category by diseases and disorders was the inflammatory response, followed by connective tissue disorders and immunological disease. The related categories by molecular function were cellular growth and proliferation, cell to cell signaling and interaction, and cellular movement.

**Figure 3:**
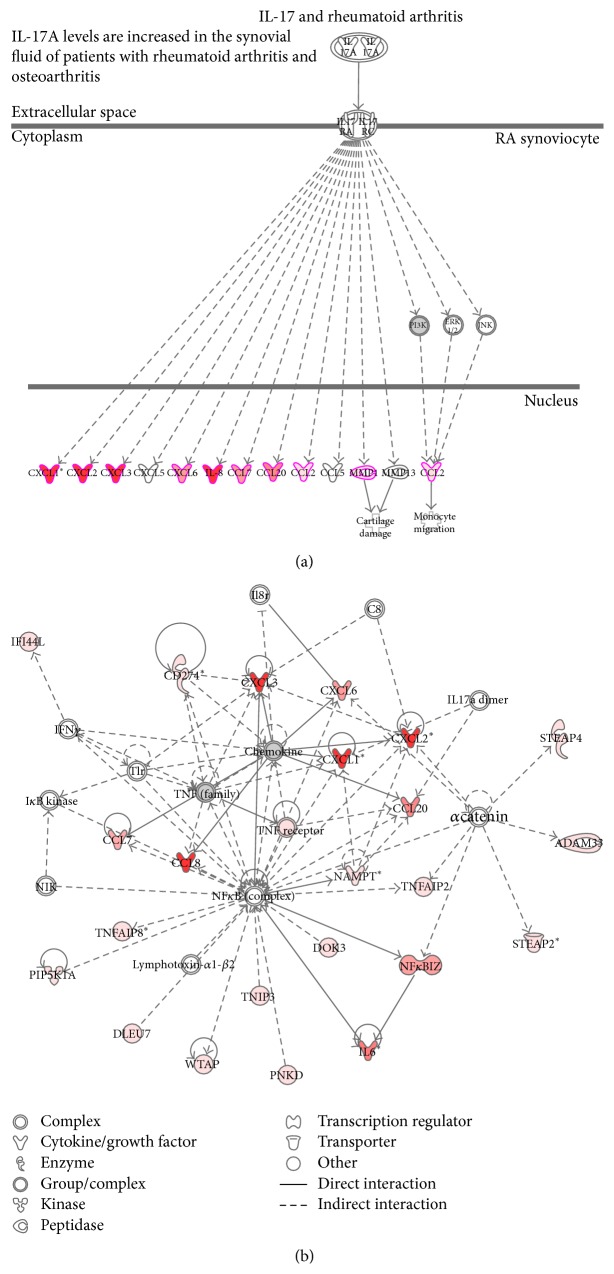
Network of the IL-17A pathway by Ingenuity Pathway Analysis (IPA). Data were analyzed using the Ingenuity Pathway Analysis system (Ingenuity System, http://www.ingenuity.com/). (a) IL-17A-induced genes associated with rheumatoid arthritis. (b) Network 1 of IL-17A-induced genes by IPA. The intensity of the node color indicates the degree of upregulation (red). Nodes are indicated by various shapes that represent the functional class of the gene product. The lines are displayed with various labels that describe the nature of the relationship between the nodes.

Next, the IL-17A-responsive genes were arranged in molecular networks using the IPA system, which linked these genes in a graphical representation of the canonical pathways. The illustration in Figure 3(a) shows the canonical pathway for “roles of IL-17A in arthritis,” which are key molecules in inflammation and destruction in arthritis. Nodes are shown as genes and/or gene products; the red nodes show genes upregulated by IL-17A in synovial fibroblasts by the microarray analysis data. The expression of numerous chemokines was upregulated by IL-17A treatment; in contrast, IL-17RA and IL-17RC were constitutively expressed and their expression in synovial fibroblasts did not change in response to IL-17A. IL-17A upregulated genes such as IL-6 and chemokines are regulated by NF*κ*B although the expression of NF*κ*B complex molecules was not responsive to IL-17A (Figure 3(b)).

### 3.4. Time Course of IL-17A-Induced Gene Expression in Synovial Fibroblasts

**Figure 4:**
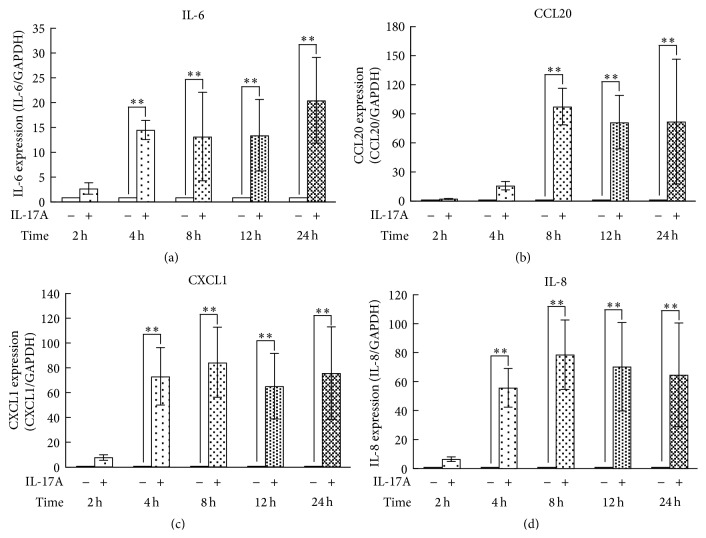
Time course of IL-17A induction of the mRNA expression of IL-6, CCL20, CXCL1, and IL-8 in synovial fibroblasts. The effect of IL-17A on (a) IL-6, (b) CCL20, (c) CXCL1, and (d) IL-8 gene expression in synovial fibroblasts was analyzed using real-time PCR following culture of the cells with or without IL-17A (10 ng/mL) for 4, 8, 12, or 24 h. Data are shown as means ± SD (*n* = 5); ^*∗∗*^ *P* < 0.01.

Signaling pathway analysis using the microarray data indicated that the expression of chemokines and IL-6 was upregulated in synovial fibroblasts by IL-17A treatment for 4 h. We therefore next analyzed the time course of IL-17A induction of the expression of these genes in synovial fibroblasts. Their expression was analyzed using real-time PCR following incubation of synovial fibroblasts with or without IL-17A for 2, 4, 8, 12, or 24 h. The gene expression of IL-6, CXCL1, and IL-8 (also called CXCL8) was significantly higher in synovial fibroblasts treated with IL-17A for 4 h to 24 h compared to nontreated control (Figures 4(a), 4(c), and 4(d)). The gene expression of CCL20 was significantly upregulated in synovial fibroblasts by IL-17A treatment for 8 to 24 h (Figure 4(b)).

### 3.5. Effect of IL-17A on IL-6 Protein Production in Synovial Fibroblasts

**Figure 5:**
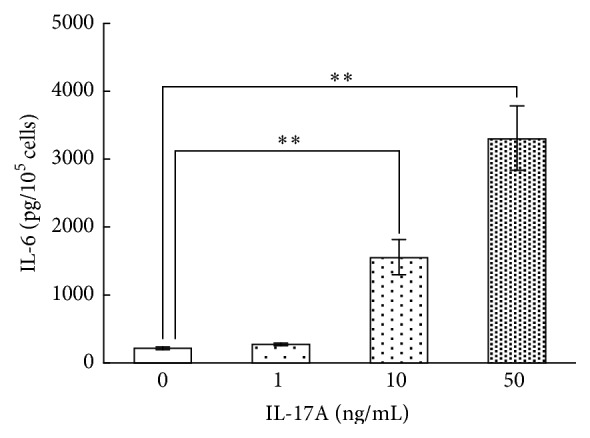
Effects of IL-17A on IL-6 protein production by synovial fibroblasts. Synovial fibroblasts were treated with the indicated concentrations of IL-17A for 24 h. The IL-6 protein levels in the conditioned medium were then assayed using ELISA. Data are shown as means ± SD (*n* = 6); ^*∗∗*^ *P* < 0.01.

**Figure 6:**
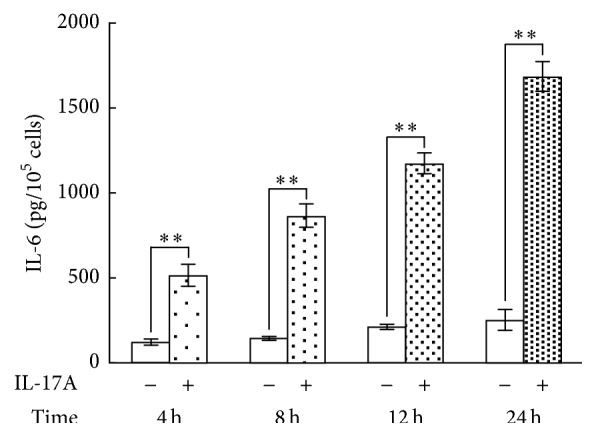
Time course of IL-17A-induced IL-6 production by synovial fibroblasts. Synovial fibroblasts were treated with 10 ng/mL IL-17A for 4, 8, 12, or 24 h. The IL-6 protein levels in the conditioned medium were then assayed using ELISA. Data are shown as means ± SD (*n* = 6); ^*∗∗*^ *P* < 0.01.

IL-6 is one of the most well-known proinflammatory cytokines implicated in the pathogenesis of various autoimmune and chronic inflammatory diseases. A number of inflammatory diseases are characterized by overproduction of IL-6. Therefore, among the IL-17A-responsive genes we selected IL-6 to examine the effect of IL-17A on protein production. Synovial fibroblasts were incubated with concentrations of IL-17A of 1, 10, and 50 ng/mL for 24 h. IL-17A increased the IL-6 levels in the conditioned media of synovial fibroblasts in a dose-dependent manner, although there was no significant difference between the cells treated with 1 ng/mL IL-17A and the untreated controls (Figure 5). We also examined the time course of IL-6 protein production in synovial fibroblasts incubated with or without 10 ng/mL IL-17A for 4, 8, 12, or 24 h. The IL-6 levels in the conditioned media were increased by IL-17A in a time-dependent manner over the entire 24 h period (Figure 6).

**Figure 7:**
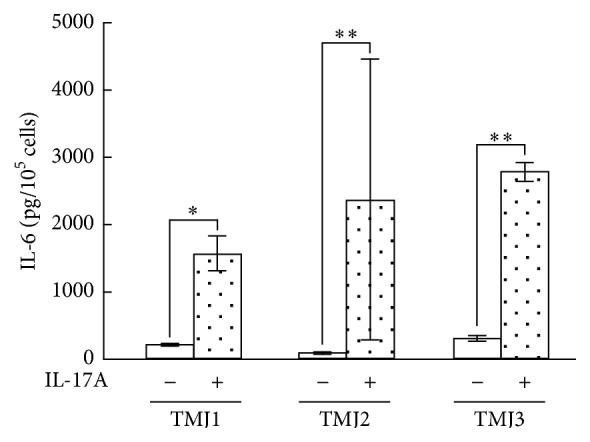
Effect of IL-17A on IL-6 production by three human synovial fibroblast samples. Synovial fibroblast samples were isolated from three patients with TMD (TMJ1-3). The cells were treated with 10 ng/mL IL-17A for 24 h, following which the IL-6 protein levels in the conditioned medium were then assayed using ELISA. Data are shown as means ± SD (*n* = 6); ^*∗*^ *P* < 0.05; ^*∗∗*^ *P* < 0.01.

In the next experiment, the effect of IL-17A on IL-6 protein production in three synovial fibroblasts samples isolated from three different patients was examined. IL-6 protein levels were significantly increased in the conditioned media from the cells treated with 10 ng/mL IL-17A for 24 h compared to the untreated control cells in all three samples, although the level of the increase varied among the individual three samples (Figure 7).

### 3.6. Effect of Signaling Inhibitors on IL-17A-Induced IL-6 Production by Synovial Fibroblasts

**Figure 8:**
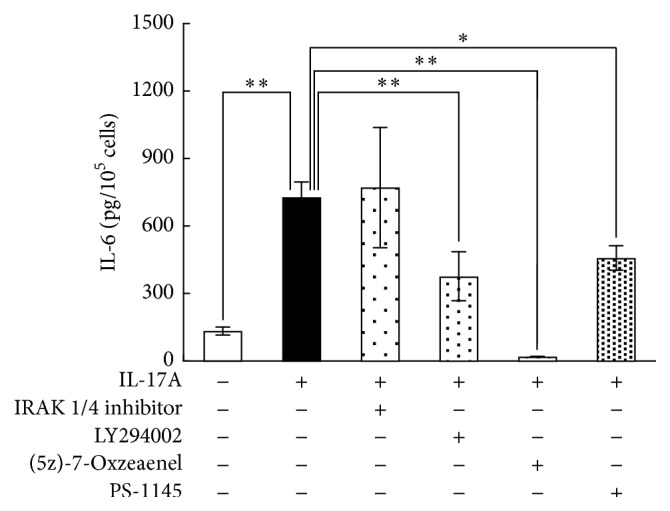
Effect of inhibitors of the NF*κ*B signaling pathway on IL-17A-induced IL-6 production by synovial fibroblasts. Synovial fibroblasts were pretreated with the IRAK 1/4 inhibitor (20 *μ*M), 20 *μ*M LY294002, 1 *μ*M (5z)-7-Oxozeaenol, or 10 *μ*M PS-1145 for 30 min and were then treated with 10 ng/mL IL-17A for 8 h, following which the IL-6 protein levels in the conditioned medium were assayed using ELISA. Results are expressed as means ± SD (*n* = 4); ^*∗*^ *P* < 0.05; ^*∗∗*^ *P* < 0.01.

The network of IPA and several previous reports suggested that cytokine expression induced by IL-17A is mediated via NF*κ*B. We therefore investigated the effects of inhibitors of the NF*κ*B signaling pathway on IL-17A-induced IL-6 production by synovial fibroblasts. The induction of IL-6 by IL-17A was decreased in synovial fibroblasts by pretreatment with LY294002 (a PI3K inhibitor), (5z)-7-Oxozeaenol (a TAK1 inhibitor), and PS-1145 (an IKK*β* inhibitor); in contrast, its production was not affected by an IRAK-1/4 inhibitor (Figure 8). IL-6 production was inhibited by 35.0% by LY294002, by 95.7% by (5z)-7-Oxozeaenol, and by 21.2% by PS-1145 (Figure 8).

## 4. Discussion

The current studies demonstrated that IL-17A plays an important role as a proinflammatory cytokine in autoimmune diseases and in chronic inflammatory diseases such as rheumatoid arthritis. To elucidate the roles of IL-17A in inflammatory progression of TMD, we isolated synovial fibroblasts from patients with TMD and examined the gene expression profiles in synovial fibroblasts treated with IL-17A. Prior to undertaking analysis of these gene expression profiles we first checked the ability of these cells to transduce IL-17 signals by confirming the expression of IL-17Rs in synovial fibroblasts; all of the IL-17 receptors were found to be expressed in synovial fibroblasts. In recent studies, a polymorphism in the IL-17RC was reported to be associated with the different splice variants observed in several cell types [25, 26]. In this study, the IL-17RC PCR product was visualized as two bands by agarose gel electrophoresis; thus there may be more than two different splicing variants of the IL-17RC in synovial fibroblasts.

Using a high throughput DNA microarray, a total of 1,710 genes showed a greater than twofold difference in expression intensity between nontreated control and IL-17A-treated synovial fibroblasts. We also investigated the biological functions and the molecular interactions of these IL-17A-responsive genes using signaling pathway analysis. Many of the responsive genes can be associated with “inflammatory response” and “immunological disease.” IL-17A upregulated the expression of numerous chemokine superfamily members that are involved in regulation of leukocyte accumulation and activation in inflammatory tissues. In this study, we examined the kinetics of the expression of CXCL1, IL-8, and CCL20 using real-time PCR. The gene expression of CXCL1 (also called Gro-*α*) and IL-8 (also called CXCL8), which are well-known chemokines, was highly upregulated by IL-17A. On the other hand, it has been reported that CCL20 (also called MIP-3*α*) functions as a chemoattractant for Th17 producing IL-17 cells [27]. The IL-17A-induced expression of CXCL1, IL-8, and CCL20 in synovial fibroblasts was maintained for 24 h. It has been reported that one key property of IL-17A is its role in orchestrating the migration of inflammatory cells, which has a central place in RA pathogenesis [28]. Since inflammatory cells have been detected in synovial tissue and fluid from patients with TMD [29, 30], IL-17A may therefore have a role in inducing inflammation such as in leukocyte attraction in TMD. In addition, the migration of Th17 cells induced by CCL20 that is produced by synovial fibroblasts may cause the increase in IL-17A levels in synovial tissue in TMD.

The mRNA expression of IL-6 was also upregulated in synovial fibroblasts by IL-17A, and its protein production was increased in IL-17A time- and dose-dependent manner. IL-17A also stimulated IL-6 protein production in all three synovial fibroblasts samples isolated from the three patients. IL-6 has an important role in inflammation and tissue destruction in joint diseases such as RA [31], and its concentration is elevated in the synovial fluids of arthritic patients [32, 33]. IL-6 was shown to have an important role in inflammation-evoked osteoclast formation and bone erosion [34]. It was recently demonstrated that IL-6 can promote Th17 cell differentiation in effector CD4+ T cell subsets [35]. This function of IL-6 is through to play a major role in the development of RA [36]. In TMD, the IL-6 level was also increased in synovial fluid from patients with ID and/or OA [22, 37]. The excessive production of IL-6 in synovial fibroblasts by IL-17A thus appears to be related to abnormalities associated with TMD.

**Figure 9:**
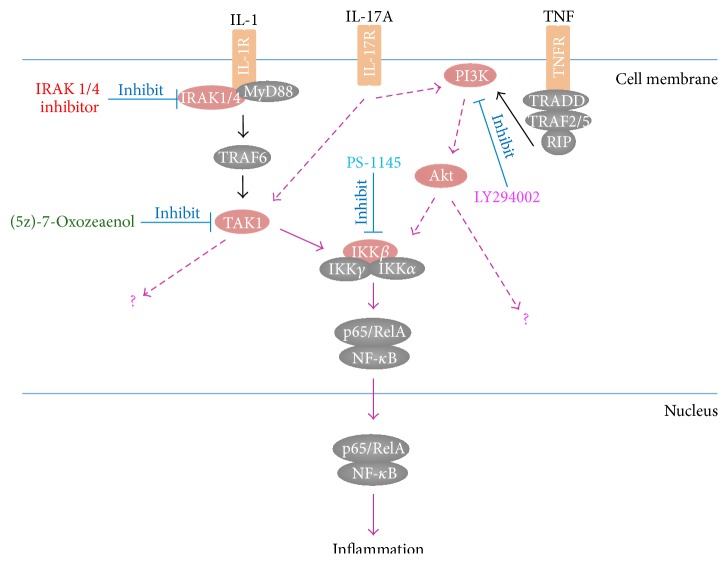
The signaling pathways of IL-17A in synovial fibroblasts. The scheme shows activation of the NF*κ*B signaling pathway through IL-17A and shared signaling with IL-1 and TNF. The purple arrows indicate signal transduction through IL-17A.

Signaling pathway analysis also indicated that the expression of chemokine and IL-6 was stimulated by NF*κ*B. IL-17A signals through a heterodimeric receptor complex of IL-17RA and IL-17RC [14], leading to activation of NF*κ*B in several cell types [38, 39]. It is generally believed that IL-17 signalingshares downstream transcription factors with IL-1*β* and TNF-*α* [40]. Previous studies have reported that IL-17 appears to exert an additive and synergistic effect with IL-1*β* and TNF-*α* as inducers of IL-6 in RA synovium [41]. We suspected that the majority of genes that were upregulated by IL-17A may be similar to those that are upregulated by IL-1*β* and TNF-*α*. We have previously investigated the gene expression profiles in TMJ synovial fibroblasts treated with IL-1*β* and/or TNF-*α* [42, 43]. To investigate IL-17A-mediated NF*κ*B activation, we examined the effects of inhibitors of NF*κ*B signaling on IL-6 production in synovial fibroblasts treated with IL-17A. We found that IL-17A-induced IL-6 production was inhibited by LY294002 (a PI3K inhibitor), (5z)-7-Oxozeaenol (a TAK1 inhibitor), and PS-1145 (an IKK*β* inhibitor) but was not affected by an IRAK-1/4 inhibitor. Therefore IL-17A signal transduction may share TAK1 and its downstream signals leading to NF*κ*B activation with IL-1*β* signal transduction. In addition, PI3K/Akt signaling, which is involved in TNF-*α*-dependent NF*κ*B activation [44], was also associated with IL-17A-induced IL-6 production by synovial fibroblasts. However, inhibition of IL-6 production by the TAK1 inhibitor was stronger than that by the IKK*β* inhibitor. It has been reported that TAK1 signaling is mediated by several other signaling transduction pathways, such as MAPK signaling pathways, in addition to NF*κ*B activation [45]. Furthermore, PI3K/Akt promotes survival by inhibiting p53 and Bak/Bax-mediated apoptosis and triggering AP1 activation [46]. We therefore suggest that IL-17A activates several signaling pathways other than the NF*κ*B activation pathway for IL-6 production in synovial fibroblasts (Figure 9).

**Figure 10:**
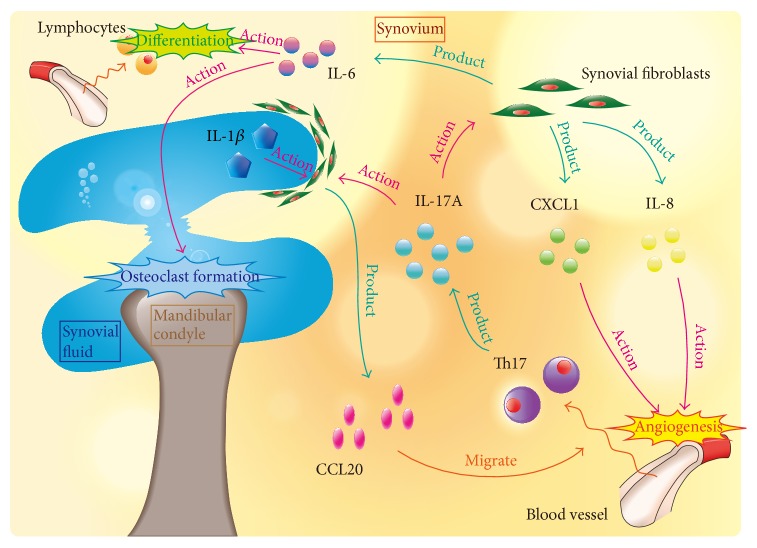
Summary of the TMJ inflammatory condition mediated by IL-17A.

In this study, our findings demonstrated that IL-17A upregulation of the expression of IL-6 and chemokines that is mediated by the NF*κ*B pathway is important in promoting leukocyte attraction to and invasion of the synovial tissue of TMD (Figure 10). We suggest that this IL-17A cascade is likely to contribute to the promotion of and to increase the inflammatory condition in TMD.

## 5. Conclusions

All IL-17 receptors are expressed in the synovial fibroblasts of TMJ. IL-17A induces the mRNA expression of chemokines and IL-6, as well as the protein production of IL-6 in synovial fibroblasts. IL-17A appears to transduce signals for IL-6 production via activation of NF*κ*B and PI3K/Akt pathways. Our data provide insights into the cellular mechanisms by which IL-17A participates in the activation of synovial fibroblasts in the inflamed temporomandibular joint.

## Figures and Tables

**Table 1:** Primers used for PCR analysis of genes.

Gene	Primers	Amplicon size (bp)
IL-6	F: 5′-AGC AAA GAG GCA CTG GCA GAA-3′	331
	R: 5′-TTG TCA TGT CCT GCA GCC ACT-3′	

CCL20 (MIP-3*α*)	F: 5′-GCA AGC AAC TTT GAC TGC TG-3′	342
	R: 5′-CAA GTC CAG TGA GGC ACA AA-3′	

CXCL1 (GRO-*α*)	F: 5′-TGC AGG GAA TTC ACC CCA AG-3′	229
	R: 5′-CAG GGC CTC CTT CAG GAA CA-3′	

IL-8 (CXCL8)	F: 5′-ACT CCA AAC CTT TCC ACC CCA-3′	129
	R: 5′-TTT CCT TGG GGT CCA GAC AGA-3′	

IL-17RA	F: 5′-TTC ATT CCT ATG CCT GAG TC-3′	204
	R: 5′-TAC AGT AAG TGG CTC GAC CT-3′	

IL-17RB	F: 5′-CCT CCG AGT AGA ACC TGT TA-3′	200
	R: 5′-GTC TGG TCT GAG TCT GGA AG-3′	

IL-17RC	F: 5′-GGA CAA ATA CAT CCA CAA GC-3′	192
	R: 5′-GAG TCA TCG GCT GAG TAG AG-3′	

IL-17RD	F: 5′-TGT GCC TTA GAG CAG GTG TG-3′	204
	R: 5′-TGT GCT TGG AAG GGA AAG TC-3′	

IL-17RE	F: 5′-GGG TCT CTC ACA TCC TGG AA-3′	207
	R: 5′-CCT CAG GAA GGG AAT GAT GA-3′	

GAPDH	F: 5′-ATC ACC ATC TTC CAG GAG-3′	318
	R: 5′-ATG GAC TGT GGT CAT GAG-3′	

IL-6, interleukin-6; CCL20, chemokine (CC motif) ligand 20; CXCL1, chemokine (CXC motif) ligand 1; IL-8, interleukin-8; IL-17R (A–E), interleukin-17 receptor (A–E); GAPDH, glyceraldehyde-3-phosphate dehydrogenase; F, forward primer; R, reverse primer.

**Table 2:** IL-17-responsive genes in synovial fibroblasts form TMJ.

Gene symbol	GenBank ID	Fold	Gene name
*Upregulated *			
Molecular function			
Chemokine activity			
CCL8	NM_005623	51.25	Chemokine (C-C motif) ligand 8
CXCL1	NM_001511	49.84	Chemokine (C-X-C motif) ligand 1 (melanoma growth stimulating activity, alpha)
CXCL2	NM_002089	38.77	Chemokine (C-X-C motif) ligand 2
CXCL3	NM_002090	35.88	Chemokine (C-X-C motif) ligand 3
CXCL8	NM_000584	24.65	Chemokine (C-X-C motif) ligand 8
CCL20	NM_004591	15.72	Chemokine (C-C motif) ligand 20
CXCL6	NM_002993	13.00	Chemokine (C-X-C motif) ligand 6
CCL7	NM_006273	11.10	Chemokine (C-C motif) ligand 7
CCL2	NM_002982	2.95	Chemokine (C-C motif) ligand 2
Cytokine activity			
AREG	NM_001657	3.32	Amphiregulin
NAMPT	AK023341	3.03	Nicotinamide phosphoribosyltransferase
BMP2	NM_001200	2.96	Bone morphogenetic protein 2
NDP	NM_000266	2.83	Norrie disease (pseudoglioma)
Cytokine receptor binding			
CSF2	NM_000758	17.62	Colony stimulating factor 2 (granulocyte-macrophage)
IL-6	NM_000600	17.19	Interleukin-6
CSF3	NM_000759	12.62	Colony stimulating factor 3 (granulocyte)
LIF	NM_002309	5.70	Leukemia inhibitory factor
IL1RN	NM_173843	2.72	Interleukin-1 receptor antagonist
IL1B	NM_000576	2.62	Interleukin-1, beta
Growth factor activity			
NTF4	NM_006179	7.22	Neurotrophin 4
NRG3	NM_001010848	5.55	Neuregulin 3
Growth factor receptor binding			
EREG	NM_001432	3.18	Epiregulin
FRS3	NM_006653	2.86	Fibroblast growth factor receptor substrate 3
FGF5	NM_033143	2.67	Fibroblast growth factor 5
G-protein coupled receptor binding			
ADORA2A	NM_000675	4.16	Adenosine A2a receptor
RTP1	NM_153708	3.37	Receptor (chemosensory) transporter protein 1
PDE4D	NM_001165899	2.17	Phosphodiesterase 4D, cAMP-specific
Receptor binding			
EPHA7	NM_004440	4.88	EPH receptor A7
ICAM4	NM_022377	2.77	Intercellular adhesion molecule 4 (Landsteiner-Wiener blood group)
STC1	NM_003155	2.73	Stanniocalcin 1
CD74	NM_001025158	2.58	CD74 molecule, major histocompatibility complex, class II invariant chain
HILPDA	NM_013332	2.30	Hypoxia inducible lipid droplet-associated
EFNB2	NM_004093	2.12	Ephrin-B2
DOK3	NM_024872	2.07	Docking protein 3
PTPN2	NM_002828	2.05	Protein tyrosine phosphatase, nonreceptor type 2
*Downregulated *			
Molecular function			
Signaling receptor activity			
HNF4A	NM_001030004	−5.42	Hepatocyte nuclear factor 4, alpha
CASS4	NM_020356	−3.21	Cas scaffolding protein family member 4
NR0B1	NM_000475	−2.51	Nuclear receptor subfamily 0, group B, member 1
NR1I3	NM_001077474	−2.05	Nuclear receptor subfamily 1, group I, member 3
Transmembrane signaling receptor activity			
TACR1	NM_015727	−97.28	Tachykinin receptor 1
CD3E	NM_000733	−66.90	CD3e molecule, epsilon (CD3-TCR complex)
OR12D2	NM_013936	−66.54	Olfactory receptor, family 12, subfamily D, member 2
OR13J1	NM_001004487	−64.29	Olfactory receptor, family 13, subfamily J, member 1
LILRB5	NM_006840	−57.98	Leukocyte immunoglobulin-like receptor, subfamily B (with TM and ITIM domains), member 5
OR52N2	NM_001005174	−54.24	Olfactory receptor, family 52, subfamily N, member 2
TAS2R40	NM_176882	−52.34	Taste receptor, type 2, member 40
TAS2R4	NM_016944	−12.98	Taste receptor, type 2, member 4
CHRNA7	NM_001190455	−9.25	Cholinergic receptor, nicotinic, alpha 7 (neuronal)
TULP1	NM_003322	−7.60	Tubby-like protein 1
